# The Stemness-High Human Colorectal Cancer Cells Promote Angiogenesis by Producing Higher Amounts of Angiogenic Cytokines via Activation of the Egfr/Akt/Nf-κB Pathway

**DOI:** 10.3390/ijms22031355

**Published:** 2021-01-29

**Authors:** Shin-Yi Chung, Ta-Chung Chao, Yeu Su

**Affiliations:** 1 Institute of Biopharmaceutical Sciences, School of Pharmaceutical Sciences, National Yang-Ming University, Shi-Pai, Taipei 11221, Taiwan; u9910417@gmail.com; 2 Department of Oncology, Division of Medical Oncology, Taipei Veterans General Hospital, Taipei 11221, Taiwan; tcchao@vghtpe.gov.tw; 3 Faculty of Medicine, School of Medicine, National Yang-Min University, Taipei 11221, Taiwan

**Keywords:** colorectal cancer stem cell, GATA6, angiogenesis, EGFR, NF-κB

## Abstract

Purpose: Cancer stem cells (CSCs) are responsible for cancer metastasis by stimulating tumor angiogenesis via various mechanisms. To elucidate the potential of the stemness-high human colorectal cancer (CRC) cells (i.e., CRCSCs) in activating angiogenesis, effects of the GATA6-overexpressing HCT-116 and HT-29 human CRC clones established previously by us in promoting the angiogenesis of human umbilical vein endothelial cells (HUVECs) were examined. Methods: Angiogenesis-promoting effects (i.e., migration, invasion, DNA synthesis, and tube formation) in HUVECs of the conditioned media (CM) from various human CRC clones were analyzed. MMP activities were assessed using a zymography assay. Western blotting and selective inhibitors were used to dissect the signaling pathway involved. IHC was used to examine the vascular density in tumor xenografts. Results: We found that the conditioned media (CM) collected from the GATA6-overexpressing clones enhanced angiogenesis of HUVECs more effectively which might be attributed partly to a higher MMP-9 production by HUVECs. Subsequently, elevated levels of IL-8 and VEGF-A were detected in the CM whose tube formation-enhancing activities were abolished by the co-treatment with either a VEGFR2 inhibitor or an IL-8 neutralizing antibody. Interestingly, increased production of these cytokines in the GATA6-overexpressing clones was due to an EGFR/AKT-mediated activation of NF-κB. Furthermore, not only were the levels of CD31 and endomucin but also the blood vessel density was much higher in the xenograft tumors grown from these clones. Conclusion: Our findings demonstrate that human CRCSCs promote a stronger angiogenesis by producing higher amounts of angiogenic factors through activation of the EGFR/AKT/NF-κB pathway.

## 1. Introduction

Colorectal cancer (CRC) is a malignancy with high incidence, morbidity, and mortality in the developed countries [1,2]. In fact, CRC is the second most common type of cancer with more than 1.8 million new cases and the disease-related mortality corresponds to about 33% worldwide per year [3,4]. Cancer stem cells (CSCs, also called tumor-initiating cells (TICs)) [5], have been shown to play important roles in tumor initiation, progression, and recurrence after therapy in many cancer types [5,6,7]. To date, numerous studies have been conducted to examine the crosstalks between CSCs and the tumor microenvironment (TME) which could support the survival, stemness, quiescence, adaption, invasion, and metastasis of the former [8,9,10,11,12]. Among various components in TME, endothelial cells (ECs) have been demonstrated to participate the formation of new blood vessels via multiple mechanisms, thereby promoting the CSC properties in colorectal cancer [13], glioblastoma [14], head and neck squamous cell carcinomas [15] and esophageal cancer [16].

Angiogenesis is a process that gives rise to new blood vessels from the pre-existing ones which consists of sequential steps including the activation of ECs, the degradation of basement membranes, the proliferation, migration, elongation then maturation of ECs through the helps of various proangiogenic factors, matrix metalloproteases (MMPs) and urokinase plasminogen activator receptor (uPAR) or cytokines [12,17,18,19,20]. Endogenous pro-angiogenic factors such as vascular endothelial growth factor-A (VEGF-A) [21], stromal derived factors 1 (SDF-1) [22], and interleukin 8 (IL-8) [23] have been extensively studied. In addition, MT1-MMP, MMP-2, and MMP-9 have also been shown as key mediators of angiogenesis and metastasis [24,25,26]. Among them, VEGF-A regulates not only angiogenesis but also vasculogenesis (i.e., the development of blood vessels from precursor cells during early embryogenesis) [27,28]. These factors act together to promote the formation of new blood vessels within tumors which then facilitate the dissemination and distant metastasis of tumor cells.

The activation of NF-κB can be divided into canonical and non-canonical pathways according to the priming factors. In brief, activation of the former begins with the stimulation by proinflammatory cytokines (e.g., TNF-α and IL-1) or Toll-like receptor ligands (e.g., LPS), leading to the phosphorylation and activation of IκB kinase (IKK) complex which in turn phosphorylates IκB, resulting in its ubiquitination and degradation, followed by the nuclear translocation of NF-κB (dimer of p65:RelA) [29,30]. Intriguingly, although no activating mutations of *NF-κB* in CRC have been reported [31], constitutive activation of NF-κB has not only been observed [32,33,34,35] but also been shown to be associated with higher tumor stage [33,36], treatment resistance [32,37,38], and poor survival outcomes [39]. In addition, NF-κB has been shown to be activated by several other pathways. For example, PI3K/AKT could also activate NF-κB by directly phopsphorylating Thr23 on IKKβ [40]. Interestingly, various growth factors could promote NF-κB activation through EFGR signaling [41]. Furthermore, the PI3K/AKT/ IKKα pathway has been reported to regulate NF-κB and β-catenin in human CRC tissues with the ability to influence transcription of the genes implicated in angiogenesis and metastasis [42].

Even though the importance of angiogenesis in the malignant progression of human CRC is well-documented, the roles of colorectal cancer stem cells (CRCSCs) in promoting this process are less well-defined. In this regard, the stable clones established previously by us from HCT-116 and HT-29 human CRC cells with enforced expression of GATA-6, a zing finger-containing transcription factor, exhibiting marked increases in the stemness properties such as the sphere- and soft agar colony-forming abilities as well as the expression levels of several CRCSC markers [43], were good stemness-high CRC models. Hence, in this study, we first examined the angiogenesis-stimulating effects of the GATA6-overexpressing clones and then elucidated its underlying mechanism.

## 2. Materials and Methods

### 2.1. Cell Culture

Human colorectal carcinoma cell lines HCT-116, HT-29 and their vector and GATA6-overexpressing clone were maintained in RPMI-1640 medium supplemented 10% fetal bovine serum (FBS), 100 units/mL penicillin, 100 µg/mL streptomycin and 25 µg/mL amphotericin B (PSA, Biological Industries, Cromwell, CT, USA) at 37 °C in 5% CO_2_. GATA6-overexpressing clone was maintained under similar conditions except that 600 µg/mL of G418 and 500 µg/mL of hygromycin were added to media in HCT-116 and HT-29 clones, respectively. Primary human umbilical vein endothelial Cells (HUVECs) were purchased from PromoCell (Heidelberg, Germany, C-12200) were grown in dishes pre-coated with 1% gelatin in Endothelial Cell Growth Medium 2 (ECGM2) (#22011, PromoCell, Heidelberg, Germany) containing 2% FCS and supplement. Passages 4 to 8 of HUVECs were routinely used in this work.

### 2.2. Preparation of Conditioned Medium

The vector-control (Vec) as well as the GATA6-overexpressing clones derived respectively from HCT-116 (OE4 and OE6) and HT-29 (OEC and OED) human CRC cells were cultured in RPMI media supplemented with 2% FBS for 48 h before their culture supernatant being collected as conditioned media (CM). The CM were then subjected to centrifugation (760× *g*) for 10 min and the supernatants were filtered through 0.22-µm filters to avoid the interference of cell debris.

### 2.3. Tube Formation Assay

Growth factor-reduced Matrigel (BD Biosciences, USA) was melted at 4 °C and added to 24-well plate (250 µL/well) and then polymerized at 37 °C for 30 min. Single cell suspension of HUVECs prepared by resuspended them into various working media which contained the CM mixed 1:1 with the ECGM2 were then seeded on the Matrigel-coated wells at a density of 4 × 10^4^ cells/well. Sixteen hours later, cells in the five chosen fields on each well were photographed under a microscope (Leica IX70, Heidelberg, Germany) and the tube length in five photographs from each well was measured by a QWin Image analysis software (Leica).

### 2.4. Invasion Assay

Transwells (polycarbonate membrane, diameter 6.5-mm, pore size 8-µm; Corning Costar, USA) coated with 0.05% gelatin (#G2500, Merck) were used to performed cell invasion assay. Briefly, HUVECs (3 × 10^4^) resuspended in 200 µL serum-free ECGM2 were seeded onto the upper chamber, and 0.6 mL of various working media were then added to the lower chamber as chemoattractants. After 16-hr incubation, the non-invading cells on the upper side of the membrane were removed by cotton swab and the invading cells on the lower side of the membrane were fixed with cold methanol and then stained with DAPI. The invading cells in three randomly selected fields were photographed under a fluorescent microscope (Leica IX70, Heidelberg, Germany) and their numbers were counted using ImageJ software.

### 2.5. Migration Assay

The stoppers were inserted into each well of a 96-well plate pre-coated with 1% gelatin before HUVECs (3 × 10^4^) being seeded into each well. After overnight incubation, the stoppers were removed and ECGM2 medium alone or different working media (0.2 mL) were added into each well. Twelve hours later, the migrated cells in the blank area were photographed by a microscope (Leica IX0, Heidelberg, Germany) and their numbers were counted using ImageJ software.

### 2.6. DNA Synthesis Assay

HUVECs were seeded into 24-wells plate (2 × 10^4^/well) and various working media (0.6 mL) were then added into each well. Eighteen hours later, 0.6 mL of BrdU (10 mg/mL, BrdU Cell Proliferation Kit, #2750; Chemicon^®^) was added into each well and after another 6-h culturing, cells were fixed by a fixing solution and the amounts of BrdU incorporated into DNA were then analyzed by incubating the fixed cells sequentially with an anti-BrdU antibody, an HRP-conjugated secondary antibody, and the substrate tetramethylbenzidine (all from Calbiochem). The absorbance at 450 nm (OD450) of each well was then measured in a spectrophotometer (TECAN Sunrise ELISA Reader).

### 2.7. Zymography Assay

Conditioned media mixed with 5× non-reducing sample buffer (125 mM Tris-HCl, 4% SDS, 0.01% bromophenol blue, 20% glycerol, pH 6.8) were then loaded onto a 7.5% acrylamide gel (0.75 mm thickness) containing gelatin. After band separation being achieved, gels were gently shaken with washing buffer (2.5% Triton X-100, 50 mM Tris HCl, 5 mM CaCl_2_, 1 µM ZnCl_2_, pH 7.5) at room temperature for 30 min twice and then soaked in the incubation buffer (1% Triton X-100, 50 mM Tris HCl, 5 mM CaCl_2_, 1 µM ZnCl_2_, pH 7.5) at 37 °C for 20 h. The gel was then incubated with staining solution (methanol:acetic acid:H_2_O = 4:1:5, 0.005% coomassie Blue) for 30 min to 1 h at room temperature with agitation before being rinsed with H_2_O until staining solution was completely removed. Finally, the gel was incubated with destaining solution (methanol:acetic acid:H_2_O = 4:1:5) until bands could clearly be seen. Zymograms were then photographed and the amount of gelatin degradation was assessed using ImageJ.

### 2.8. Western Blotting

For total lysate preparation, cells were washed with cold PBS and scraped into 1 mL cold PBS. After centrifugation, cells were lysed in RIPA buffer [50 mM Tris-HCl, 150 mM NaCl, 0.1% sodium dodecyl sulfate (SDS), and 1% Nonidet P-40 (NP-40); pH 7.4]. To prepare nuclear fraction, cells were incubated first in a low-salt buffer (10 mM Tris-HCl, 10 mM NaCl, 3 mM MgCl_2_, 0.5% NP-40; pH 7.4) and nuclei were then pelleted by centrifugation. Nuclear proteins were subsequently extracted with a high-salt buffer (20 mM HEPES, 25% glycerol, 0.4 M NaCl, 1.5 mM MgCl2, 0.2 mM EDTA; pH 7.9) on ice. Total lysates or nuclear proteins (20 µg) were separated on a 10% SDS-polyacrylamide gel and processed for immunoblotting with the primary antibodies against p-IκB (Abcam #ab133462), total IκB(Abcam #ab32518), p-NF-κB p65 (Cell Signaling #3033), total NF-κB p65 (Cell Signaling #4764), and NF-κB p105/50 (Abcam #ab32360), total AKT (Cell Signaling #2938), p-AKT (Cell Signaling #4060), total EGFR (Cell Signaling #4267), p-EGFR (Cell Signaling #3777), and lamin (MDBio #AB0234). After overnight incubation at 4 °C, the blots were washed several times with 1X TBST (Tris-buffered saline-Tween 20) before being probed with the horseradish peroxidase-conjugated secondary antibodies. Signals were detected using an enhanced chemiluminescence system (ECL; NEN Life Science, Boston, MA, USA) and their intensities were quantified by densitometry (ImageJ).

### 2.9. Quantitative RT-PCR

Total RNAs (5 µg) isolated from cells using TRIzol reagent (MDBio, Inc., Taipei, Taiwan) were reverse transcribed using MMLV RT (Thermo Fisher Scientific Waltham, MA, USA). SYBR Green-based quantitative PCR analysis was then carried using the CFX Connect™ Real-Time PCR Detection System (Bio-Rad) with primer sets designed to analyze the expression of specific genes including *MMP2* (forward: 5′-ATGACAGCTGCACCACTGAG-3′ and reverse: 5′-ATTTGTTGCCCAGGAAAGTG-3′), *MMP9* (forward: 5′-TTGACAGCGACAAGAAGTGG-3′ and reverse: 5′-GCCATTCACGTCGTCCTTAT-3′), *VEGF-A* (forward: 5′-GGGCAGAATCATCACGAAGT-3′ and reverse: 5′-TGGTGATGTTGGACTCCTCA-3′), *ANG1* (forward: 5′-GGCGTTTTGTTGTTGGTCTT-3′ and reverse: 5′-TGATGTCTTTGCAGGGTGAG-3′), *SDF-1* (forward: 5′-TCAGCCTGAGCTACAGATGC-3′ and reverse: 5′-CTTTAGCTTCGGGTCAATGC-3′), *THBS1* (forward: 5′-TTGTCTTTGGAACCACACCA-3′ and reverse: 5′-CTGGACAGCTCATCACAGGA-3′) *IL-1β*(forward: 5′-AGCTGAAAGCTCTCCACCTC-3′ and reverse: 5′-TTGGGATCCACACTCTCCAG-3′), *IL-6* (forward: 5′-GAGTCACAGAAGGAGTGGCT-3′ and reverse: 5′-GACCACAGTGAGGAATGTCC-3′), *IL-8* (forward: 5′-ATGACTTCCAAGCTGGCCGTGGCT-3′ and reverse: 5′-TCTCAGCCCTCTTCAAAAACTTCTC-3′), *TNF-α*(forward: 5′-CCACCACGCTCTTCTGTCTA-3′ and reverse: 5′-TCCCTTGAAGAGAACCTGGG-3′), and *M-CSF* (forward: 5′-CTCTGTCTCCCCTCATCAGC-3′ and reverse: 5′-TCCTTGACAACTGGGGTCTC-3′). The reaction conditions were: 95 °C for 10 min, 40 cycles at 95 °C for 30 s and 65 °C for 30 s, and 72 °C for 30 s. The relative quantity of target gene expression was calculated using the comparative Ct method (ΔΔCt), which was normalized to endogenous GAPDH levels using CFX Manager version 3.1 (BioRad).

### 2.10. Immunohistochemical Staining

Xenotransplantation of various human CRC clones onto nude mice was carried out primarily as described previously [43]. Immunohistochemical (IHC) staining of the tissue sections cut from tumors grown from the wild-type HCT-116 and HT-29 cells as well as the GATA6-overexpressing clones derived respectively from them was performed using the NovolinkTM Max polymer detection system (#RE7260-CE, Leica). Briefly, the formalin-fixed, paraffin-embedded tumor tissues harvested from the nude mice transplanted with the clones were sectioned, placed on slides, and de-paraffined in xylene for 5 min. Tissue sections were then gradually hydrated through graded alcohol (100%, 90%, 85%, 70%, and 50%) washes and subjected to epitope retrieval (#S1699, Dako, Santa Clara, CA, USA) prior to staining. For CD31 staining, a polyclonal anti-CD31 antibody (1:200, #BD550274) and an HRP-conjugated anti-rabbit antibody were used as primary and secondary antibodies, respectively. A similar protocol was applied for endomucin (1:100, #ab106111) staining. All slides were counterstained with hematoxylin, mounted by flouromount-G (SouthernBiotech #0100-01) and then photographed under a microscope (Olympus BX43)

### 2.11. Statistical Analysis

All data are expressed as mean ± SD of three independent experiments. Experimental groups were compared for statistical significance using a one-way ANOVA with the LSD post hoc test and different letters (a, b, c etc) represent different levels of significance (*p* < 0.05). A *p*-value < 0.05 was statistically significant by Student’s *t*-test.

## 3. Results

### 3.1. Conditioned Media Collected from the GATA6-Overexpressing Human CRC Cells Induce Angiogenesis More Effectively

**Figure 1:**
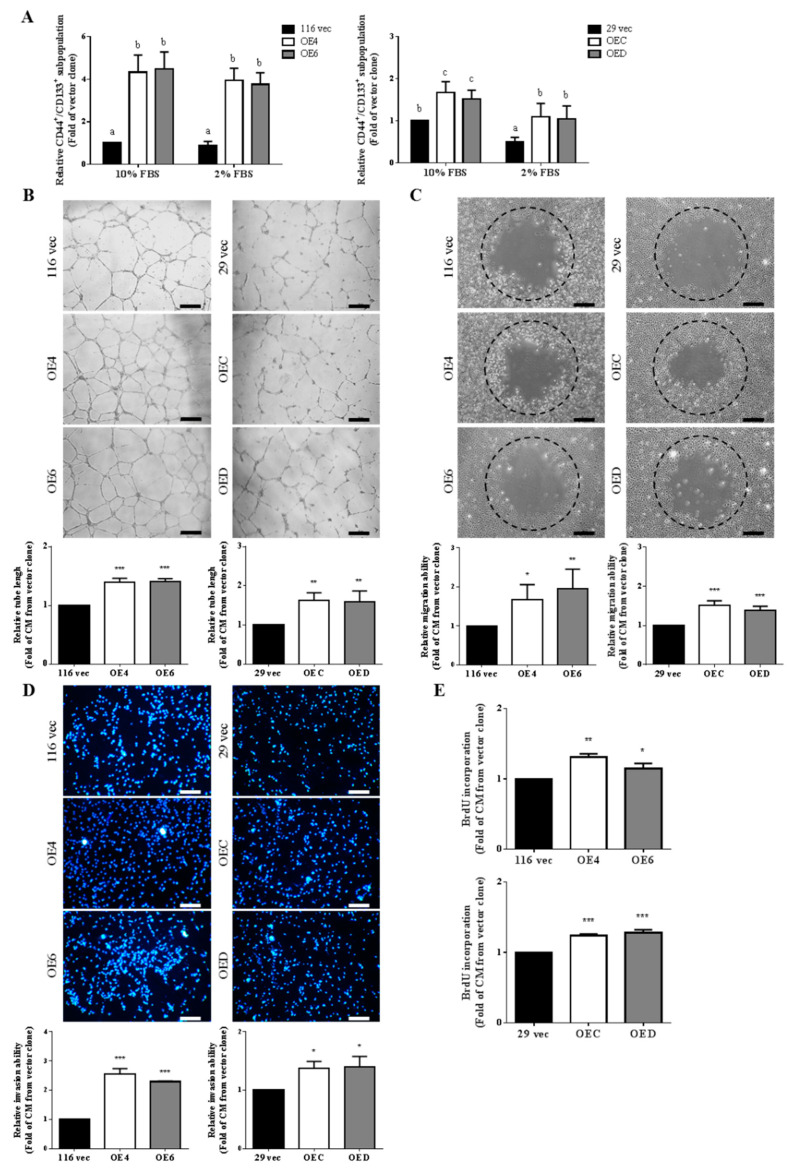
Conditioned media (CM) collected from stemness-high human colorectal cancer cells induce a stronger angiogenesis of endothelial cells. (**A**) The percentages of CD44^+^/CD133^+^ subpopulations in the vector and the GATA6-overexpressing HCT-116 (116 vec, OE4, and OE6) (left) as well as HT-29 (29 vec, OEC, and OED) (right) clones after being cultured in the media containing 10 and 2% FBS, respectively, for 48 h were measured by flow cytometry after cells were incubated simultaneously with the FITC-conjugated anti-CD44 and PE-conjugated anti-CD133 antibodies. Data (mean ± SD, N = 3) were analyzed by one-way ANOVA with the LSD post hoc test and different letters (a–c) represent different levels of significance (*p* < 0.05). (**B**) HUVECs were suspended in the working media prepared as described in the “Materials and methods” using CM collected respectively from the HCT-116 (left) as well as the HT-29 clones (right) and added into 24-well plates pre-coated with Matrigel. The tube structure was examined at 16 h after cell seeding and the length of vessels in five randomly chosen fields was counted by using the QWin software. Scale bar is 100 µm. The quantitative results are the mean  ±  SD of three independent experiments. (**C**) HUVECs were seeded into 96-well plate pre-coated with 1% gelatin which contained a stopper. The media were replaced by the CM collected respectively from the HCT-116 (left) as well as the HT-29 clones (right). Twelve hours later, the areas covered by cells within the circles were measured by Image J. Scale bar is 100 µm. (**D**) HUVECs (3 × 10^4^/well) were seeded onto transwell pre-coated with 0.05% gelatin. Once the cells attached, the media in the lower chambers were replaced by the CM collected respectively from the HCT-116 (left) as well as the HT-29 clones (right). Sixteen hours later, cells migrated to the bottom side of the filter were stained with DAPI and counted by Image J. Scale bar is 100 µm. (**E**) HUVECs (2 × 10^4^/well) were seeded onto 24-well plate pre-coated with 1% gelatin Once the cells attached, the media were replaced by the CM collected respectively from the HCT-116 (upper) as well as the HT-29 clones (lower). Eighteen hours later, cells were incubated with 10 mg/mL BrdU for 6 h and its incorporation was then detected as described in the “Materials and methods”. (**B**–**E**) Data are the mean  ±  SD of three independent experiments. * *p* < 0.05, ** *p* < 0.01, and *** *p* < 0.005 compared with the respective vector clones by Student’s *t*-test.

To investigate whether the stemness-high human colorectal cancer (CRC) cells could induce a stronger angiogenesis of endothelial cells (ECs), we first prepared the conditioned media (CM) from the vector-control as well as the stemness-high GATA6-overexpressing clones established previously from HCT-116 (116 Vec, OE4, and OE6) and HT-29 (29 Vec, OEC, and OED) human CRC cells [43] whose GATA6 levels were 4- and 5-folds higher than their respective control clones (Figure S1), by collecting the media containing 2% FBS used to culture these cells for 48 h. To assure that the stemness properties of the GATA6-overexpressing clones were minimally affected by a short-term incubation in low serum, flow cytometry was used to compare the CD133^+^/CD44^+^ stem cell-like subpopulations in the six clones cultured respectively in the media containing 10 or 2% FBS for 48 h. As can be seen, the double positive subpopulations in the GATA6-overexpressing HCT-116 clones were not affected at all by short-term incubation in low serum (Figure 1A, upper panel), while those in the HT-29 OEC and OED clones were markedly decreased but nonetheless remained significantly higher than that of the vector clone (Figure 1A, lower panel). Having found that the stemness properties of the GATA6-overexpressing clones were negligibly affected by short-term low serum treatment, tube formation assays were then performed to compare the effects of the CM collected from these clones on the angiogenesis of human umbilical vein endothelial cells (HUVECs). As shown in Figure 1B, the CM collected from the GATA6-overexpressing clones induced much stronger tube formation of HUVECs. The effects of the CM on the migration and invasion abilities of HUVECs were next examined. To no surprise, both the migration (Figure 1C) and invasion (Figure 1D) abilities of HUVECs increased by the CM from the GATA6-overexpressing clones were significantly higher than those from the vector-control clones. In addition, higher levels of DNA synthesis in HUVECs were also detected after being treated with the CM collected from the GATA6-overexpressing clones (Figure 1E). Together, these results suggested that CM collected from the stemness-high HCT-116 and HT-29 human CRC cells induce a stronger in vitro angiogenesis of HUVECs.

### 3.2. CM Collected from the GATA6-Overexpressing Clones Increase Both the mRNA Levels and Activities of MMP-9 in HUVECs

**Figure 2:**
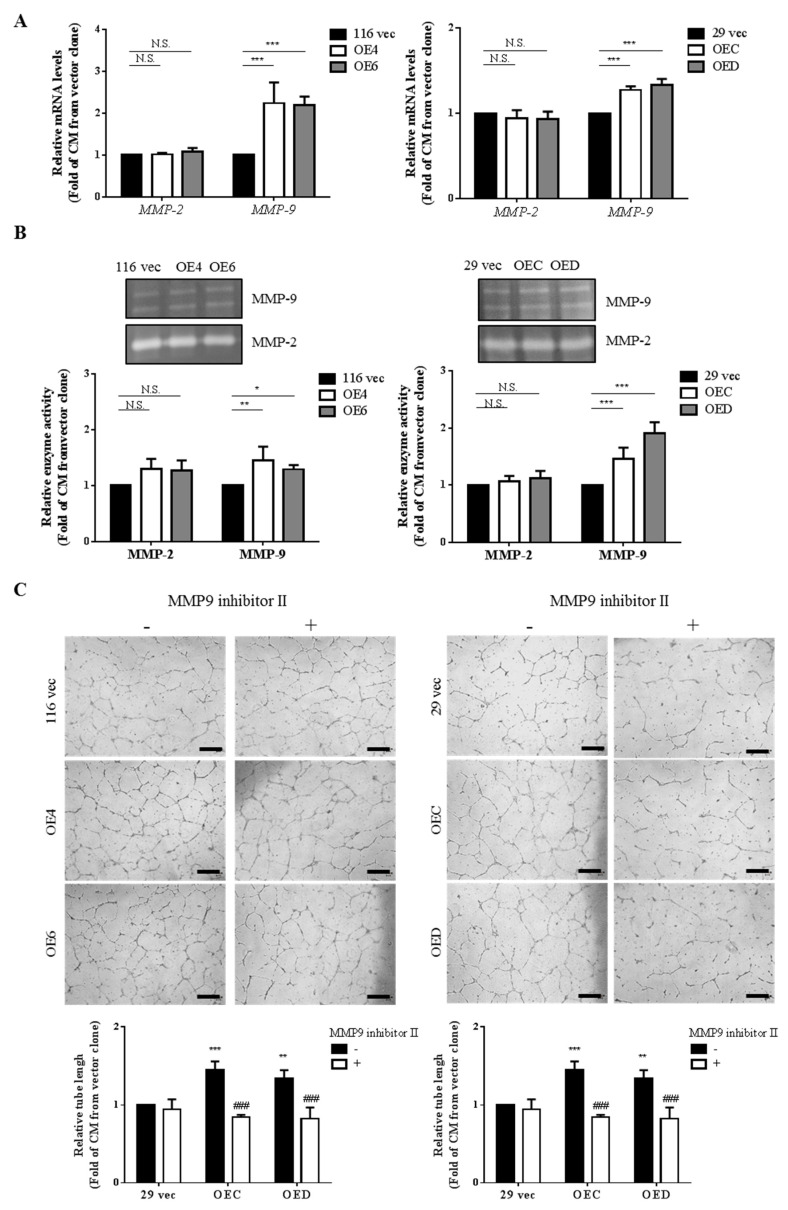
CM collected from the GATA6-overexpressing clones increase both the mRNA levels and activities of MMP-9 in HUVECs and the latter is critical for their tube formation. (**A**) Total RNAs (5 µg) isolated from HUVECs after being cultured in the CM collected respectively from the 116 vec, OE4, and OE6 as well as the 29 vec, OEC, and OED clones for 48 h were subjected to RT-qPCR analyses to determine the mRNA levels of *MMP-2* and *-9* genes. Data are the mean  ±  SD of three independent experiments. *** *p* < 0.005 compared with the respective vector clones by Student’s *t*-test. N.S. indicates no significant difference compared also with the respective vector clones. (**B**) The activities of MMP-2 and MMP-9 released from HUVECs after being cultured in the CM collected respectively from the HCT-116 (left) as well as the HT-29 (right) for 24 h were examined by gelatin zymography as described in the “Materials and methods”. The quantitative results shown in the bar graphs (lower panels) are the mean  ±  SD of three independent experiments. * *p* < 0.05, ** *p* < 0.01, and *** *p* < 0.005 compared with the respective vector clones by Student’s *t*-test. (**C**) Tube formation assays of HUVECs were carried out primarily as described above using the CM collected from three clones derived respectively from HCT-116 and HT-29 cells without or with MMP9 inhibitor II (1 µM). The quantitative results shown in the lower panels are the mean  ±  SD of three independent experiments. * *p* < 0.05, ** *p* < 0.01, and *** *p* < 0.005 compared with the respective untreated vector clones by Student’s *t*-test. ### *p* < 0.005 compared with the corresponding untreated groups by Student’s *t*-test.

Several studies indicated that matrix metalloproteinases (MMPs) such as MMP-2 [44], MMP-7 [45] and MMP-9 [46] play important roles in the migration of ECs [47] and even angiogenesis of various cancers [48] by degrading extracellular matrix (ECM) and releasing various growth factors [49]. To examine the possible involvement of MMPs in the stronger angiogenesis-promoting effects of the CM collected from the GATA6-overexpressing clones, the mRNA levels of MMP-2 and -9 in HUVECs after being treated with the CM collected from various HCT-116 and HT-29 clones for 48 h were analyzed by RT-qPCR. As can be seen in Figure 2A, the MMP-9 but not MMP-2 mRNA levels in HUVECs were significantly increased by the treatment of the CM collected from the GATA6-overexpressing clones. Subsequently, the enzyme activities of MMP-2 and -9 in the culture media of HUVECs were analyzed by gelatin zymography. In agreement with the above results, only MMP-9 activities in the culture media of HUVECs were markedly enhanced by the CM from the GATA6-overexpressing clones (Figure 2B). Furthermore, the tube formation abilities of HUVECs were dramatically decreased by the addition of 1 µM MMP9 inhibitor II in the CM from the GATA6-overexpressing clones used in these assays (Figure 2C). Our results demonstrated that higher production of MMP-9 by HUVECs induced by the CM from the GATA6-overexpressing clones is at least partially responsible for their angiogenesis-enhancing effects.

### 3.3. Examination of the Expression Levels of Various Angiogenic Factors and Cytokines in the Aforementioned Clones as Well as in the CM Collected from Them

**Figure 3:**
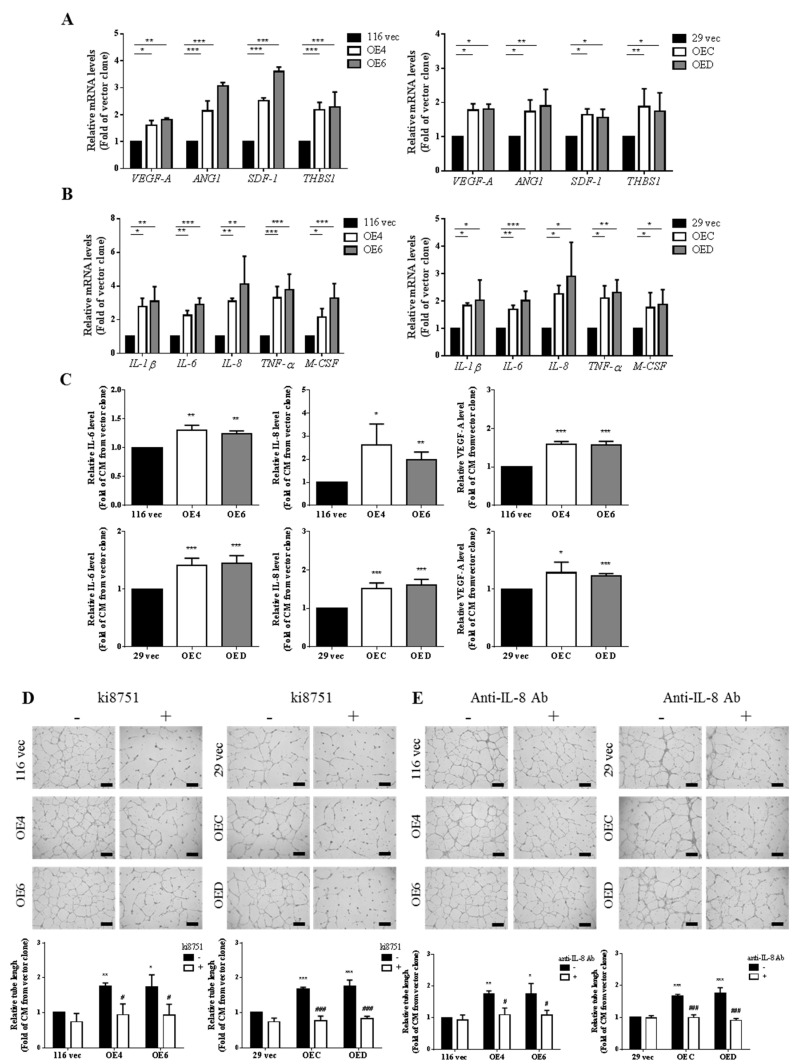
Both VEGF-A and IL-8 are the key proangiogenic factors produced by the GATA6-overexpressing human CRC clones. (**A**) The mRNA levels of various pro-angiogenic factor genes including *VEGF-A*, *ANG1*, *SDF-1*, and *THBS1* in the vector as well as the GATA6-overexpressing HCT-116 (left) and HT-29 (right) clones were analyzed by RT-qPCR using total RNAs isolated from them. (**B**) The mRNA levels of several cytokines genes including *IL-1β, IL-6*, *IL-8*, *TNF-α*, and *M-CSF* in the vector as well as the GATA6-overexpressing HCT-116 (left) and HT-29 (right) clones were examined by RT-qPCR as above described. (**C**) The levels of IL-6, IL-8, and VEGF-A in the CM collected respectively from the 116 vec, OE4, and OE6 clones (upper panels) as well as the 29 vec, OEC, and OED clones (lower panels) were analyzed by ELISA. Tube formation assays of HUVECs were carried out primarily as described above using the CM collected from three clones derived respectively from HCT-116 and HT-29 cells except that (**D**) 2 nM ki8751, a small-molecule VEGFR2 inhibitor, or (**E**) 0.1 µg/mL of IL-8 neutralizing antibody were added into half of the samples (right panels). (**A**–**C**) Data are the mean  ±  SD of three independent experiments. * *p* < 0.05, ** *p* < 0.01, and *** *p* < 0.005 compared with the respective vector clones by Student’s *t*-test. (**D**,**E**) The quantitative results shown in the lower panels are the mean  ±  SD of three independent experiments. * *p* < 0.05, ** *p* < 0.01, and *** *p* < 0.005 compared with the respective untreated vector clones by Student’s *t*-test. # *p* < 0.05 and ### *p* < 0.005 compared with the corresponding untreated groups by Student’s *t*-test.

To identify the components released by the GATA6-overexprssing clones that are responsible for enhancing the angiogenesis of HUVECs, the mRNA levels of several angiogenic factors (e.g., vascular endothelial growth factor A (VEGF-A), angiopoietin 1 (ANG1), and stromal cell-derived factor 1 (SDF-1)), a pro-metastatic factor (i.e., thrombospondin 1 (THBS1)), as well as some cytokines (e.g., IL-1β, IL-6, IL-8, tumor necrosis factor alpha (TNF-α), and macrophage colony-stimulating factor (M-CSF)) in these cells were next analyzed by RT-qPCR. Indeed, higher mRNA levels of these genes were detected in both GATA6-overexpressing HCT-116 and HT-29 clones (Figure 3A,B). In accordance, much higher levels of IL-6, IL-8, and VEGF-A were also found in the CM collected from these clones (Figure 3C). Together, above findings suggested that the stemness-high human CRC cells produced higher number of proangiogenic cytokines (e.g., VEGF-A and IL-8).

### 3.4. VEGF-A and IL-8 are the Main Proangiogenic Factors Present in the CM Collected from the GATA6-Overexpressing Clones

In addition to VEGF-A, IL-8 has also been shown to enhance the survival and proliferation as well as trigger microvascular formation of ECs by interacting with its receptors CXCR1 and CXCR2 [50,51,52]. To examine the roles of these two cytokines in the angiogenesis-promoting effects of the CM collected from the GATA6-overexpressing clones, tube formation assays of the CM were carried out in the absence and presence of ki8751, a small-molecule VEGFR2 inhibitor, or an IL-8-neutralizing antibody. As can be seen, the angiogenesis-promoting effects of the CM collected from the GATA6-overexpressing clones were dramatically suppressed by the addition of either ki8751 (Figure 3D) or the IL-8-neutralizing antibody (Figure 3E), suggesting that VEGF-A and IL-8 are the main proangiogenic factors present in the GATA6-overexpressing human CRC clones.

### 3.5. Higher Production of VEGF-A and IL-8 from the GATA6-Overexpressing Human CRC Cells Is Due to a Hyperactivation of the NF-κB Pathway

**Figure 4:**
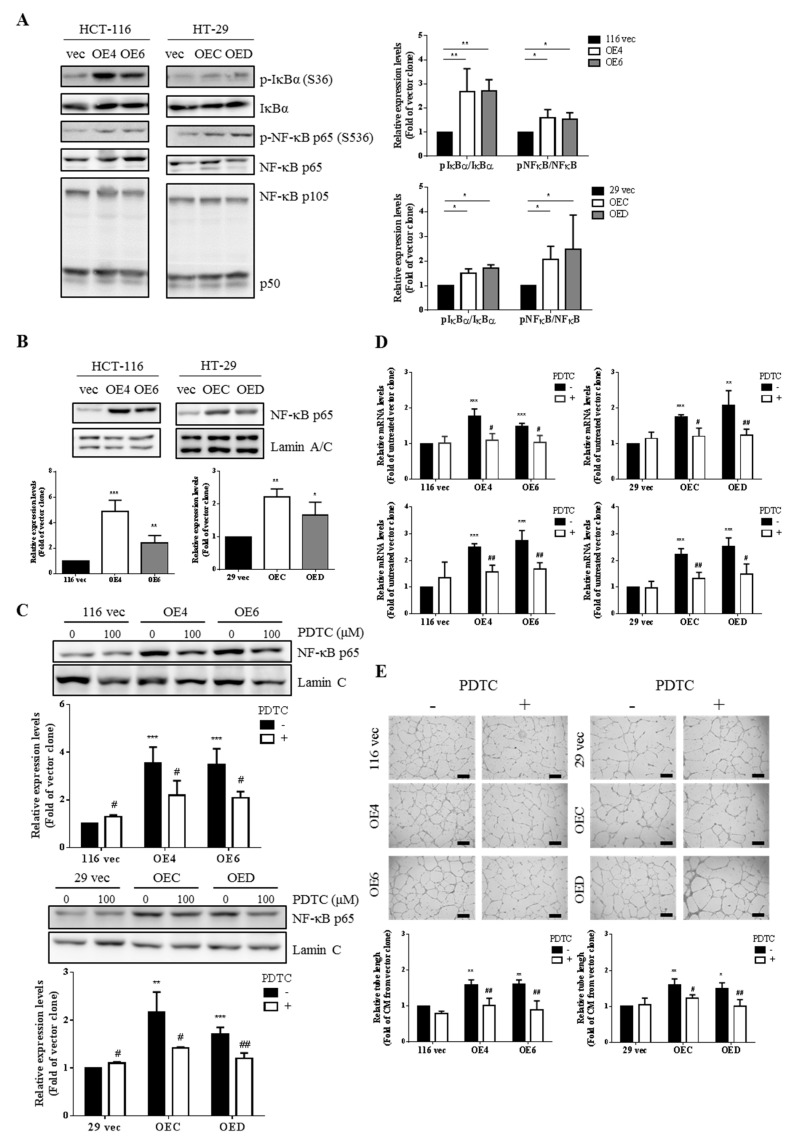
Activation of the NF-κB pathway in the GATA6-overexpressing clones is responsible for higher production of VEGF-A and IL-8 by them. (**A**) Total lysates (20 µg) prepared from three clones derived respectively from HCT-116 and HT-29 cells were subjected to western blot analyses using primary antibodies against p-IκB (ser36), total IκB, p-NF-κB p65 (ser536), total NF-κB p65, and NF-κB p105/50 as probes, respectively. The quantitative results (right panels) obtained by densitometry are the mean  ±  SD of three independent experiments. * *p* < 0.05 and ** *p* < 0.01 compared with the respective vector clones by Student’s *t*-test. (**B**) Nuclear fractions (20 µg) prepared from three clones derived respectively from HCT-116 and HT-29 cells were subjected to western blotting. Lamin C signals were used as nuclear loading controls. The quantitative results (bar graphs) obtained by densitometry are the mean ± SD of three independent experiments. * *p* < 0.05 and ** *p* < 0.01 compared with the respective vector clones by Student’s *t*-test. (**C**) Nuclear fractions (20 µg) prepared from three clones derived respectively from HCT-116 (upper panels) and HT-29 (lower panels) cells after they were treated without or with 100 µM PDTC for 24 h were subjected to western blotting to examine the nuclear levels of NF-κB p65. Lamin C signal was used as a nuclear loading control. The quantitative results (bar graphs) obtained by densitometry are the mean ± SD of three independent experiments. ** *p* < 0.01 and *** *p* < 0.005 compared with the respective vector clones by Student’s *t*-test. # *p* < 0.05 and ## *p* < 0.01 compared with the respective untreated groups. (**D**) Total RNAs (5 µg) isolated from the aforementioned clones after being treated without or with 100 µM PDTC for 24 h were subjected to RT-qPCR analyses to determine the mRNA levels of VEGF-A (upper panels) and IL-8 (lower panels). Data are the mean  ±  SD of three independent experiments. ** *p* < 0.01 and *** *p* < 0.005 compared with the respective vector clones by Student’s *t*-test. # *p* < 0.05 and ## *p* < 0.01 compared with the corresponding untreated groups. (**E**) Tube formation assays of HUVECs were carried out primarily as described above using the CM collected from the clones treated without or with PDTC before their collection. * *p* < 0.05 and ** *p* < 0.01 compared with the respective untreated vector clones by Student’s *t*-test. # *p* < 0.05 and ## *p* < 0.01 compared with the corresponding untreated groups by Student’s *t*-test.

Since the expression of both *VEGF-A* and *IL-8* genes has been shown to be activated simultaneously by various transcription factors including nuclear factor-kappa B (NF-κB) [53,54], we hence examined whether the NF-κB pathway was more active in the GATA6-overexpressing clones by western blotting. Indeed, higher levels of phosphor-IκB and phosphor-NF-κB p65 but not NF-κB p50 were detected in the GATA6-overexpressing clones (Figure 4A). In accordance, higher nuclear levels of NF-κB p65 were also found in these clones (Figure 4B). To further elucidate the role of NF-κB in the increased production of VEGF-A and IL-8 from the GATA6-overexpressing clones, we analyzed the effect of a selective NF-κB inhibitor, pyrrolidine dithiocarbamate (PDTC), and found that it significantly diminished the nuclear levels of NF-κB (Figure 4C). The mRNA levels of *VEGF-A* and *IL-8* in the GATA6-overexpressing clones treated without or with PDTC were subsequently analyzed by RT-qPCR. As can be seen in Figure 4D, the increased mRNA levels of these cytokine genes in the clones were dramatically reduced after being treated with PDTC. We next assessed whether this NF-κB inhibitor could abolish the angiogenesis-promoting activities of the CM collected from the GATA6-overexpressing clones. As expected, our results indicated that the tube formation-stimulatory effects of CM prepared from the clones were severely reduced when these cells were treated with PDTC before CM collection (Figure 4E). Collectively, our findings strongly suggested that GATA6 overexpression in human CRC cells results in a NF-κB-mediated increased production of two angiogenic factors, VEGF-A and IL-8, which in turn enhance the angiogenesis of ECs.

### 3.6. EGFR/AKT Pathway Is Responsible for NF-κB Activation in the GATA6-Over-Expressing Human CRC Clones

**Figure 5:**
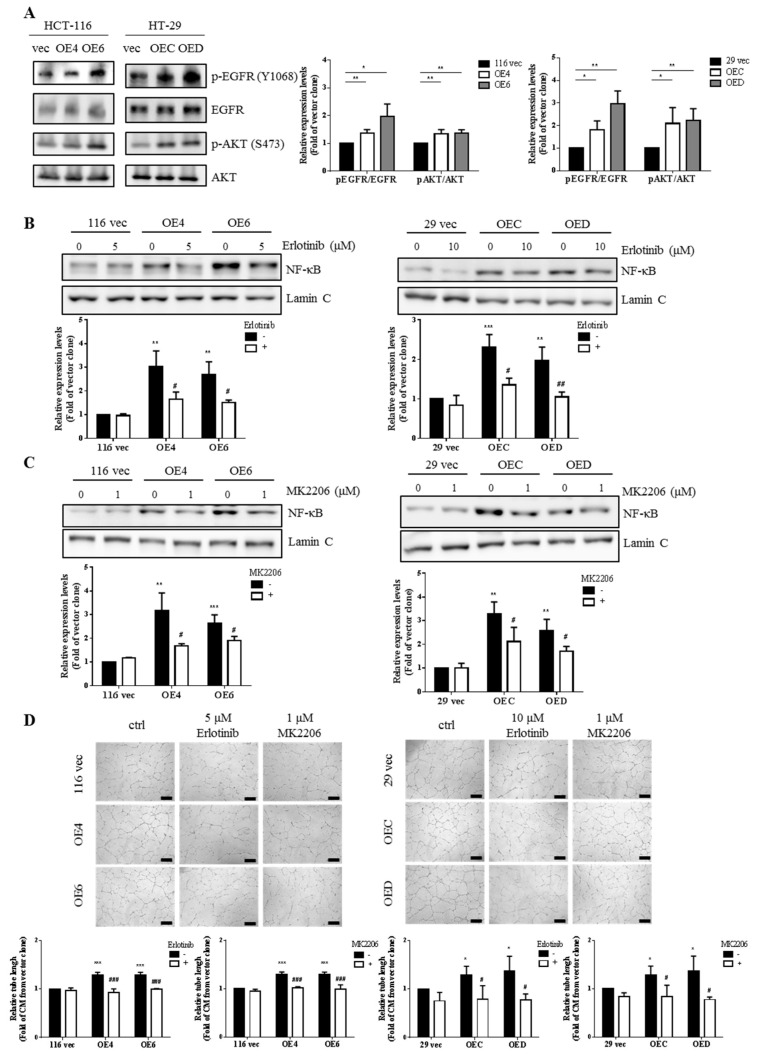
The EGFR/AKT pathway is responsible for the activation of NF-κB in the GATA6-overexpressing human CRC clones. (**A**) Total lysates (20 µg) prepared from three clones derived respectively from HCT-116 and HT-29 cells were subjected to western blot analyses using primary antibodies phospho-EGFR (Y1068), total EGFR, phospho-AKT (S473), and total AKT as probes, respectively. The quantitative results (bar graphs) obtained by densitometry are the mean ± SD of three independent experiments. * *p* < 0.05 and ** *p* < 0.01 compared with the respective vector clones by Student’s *t*-test. Nuclear fractions (20 µg) prepared from the aforementioned clones after they were treated without or with 5 or 10 µM erlotinib (**B**) and without or with 1 µM MK2206 (**C**), respectively, for 24 h were subjected to western blotting to examine the nuclear levels of NF-κB p65. Lamin C signal was used as a nuclear loading control. The quantitative results obtained by densitometry (the lower panels). The quantitative results (bar graphs) were obtained by densitometry. (**D**) Tube formation assays of HUVECs were carried out primarily as described above except that cells were treated respectively without or with 5 or 10 µM erlotinib as well as without or with 1 µM MK2206 before the CM being collected. The quantitative results in (**B**–**D**) (bar graphs) are the mean ± SD of three independent experiments. * *p* < 0.05, ** *p* < 0.01, and *** *p* < 0.005 compared with the respective untreated vector clones by Student’s *t*-test. # *p* < 0.05, ## *p* < 0.01, and ### *p* < 0.005 compared with the corresponding untreated groups by Student’s *t*-test.

Since several studies have already demonstrated that CD44 could be both a co-regulator and a downstream target of EGFR signaling [55,56] plus EGFR-induced AKT could activate NF-κB in HCT-8 human CRC cells [57], we postulated that EGFR signaling might be more active in the stemness-high GATA6-overexpressing human CRC clones. Western blotting was next performed to investigate this possibility and significantly higher levels of phosphor-EGFR (Y1068) and phosphor-AKT (S473) were found in the GATA6-overexpressing clones (Figure 5A), suggesting that the EGFR/AKT pathway was more active in these cells. Subsequently, we analyzed the nuclear levels of NF-κB in the clones treated without and with erlotinib, a specific EGFR inhibitor, and MK2206, an AKT inhibitor, to examine whether the EGFR/AKT pathway was responsible for NF-κB activation. As can be seen, marked decreases in the nuclear levels of NF-κB p65 by the respective treatments with erlotinib (Figure 5B) and MK2206 (Figure 5C) were detected only in the GATA6-overexpressing clones. In accordance, the tube formation-stimulatory effects of the CM prepared from the clones were completely abolished by the respective additions of erlotinib and MK2206 before their collection (Figure 5D). Taken together, our results strongly suggest that hyperactivation of the EGFR/AKT/NF-κB pathway is essential for the overproduction of various proangiogenic factors by the GATA6-overexpressing human CRC clones.

### 3.7. Stronger Angiogenesis Is Observed in the Tumor Xenografts Grown from the GATA6-Overexpressing Human CRC Clones

**Figure 6:**
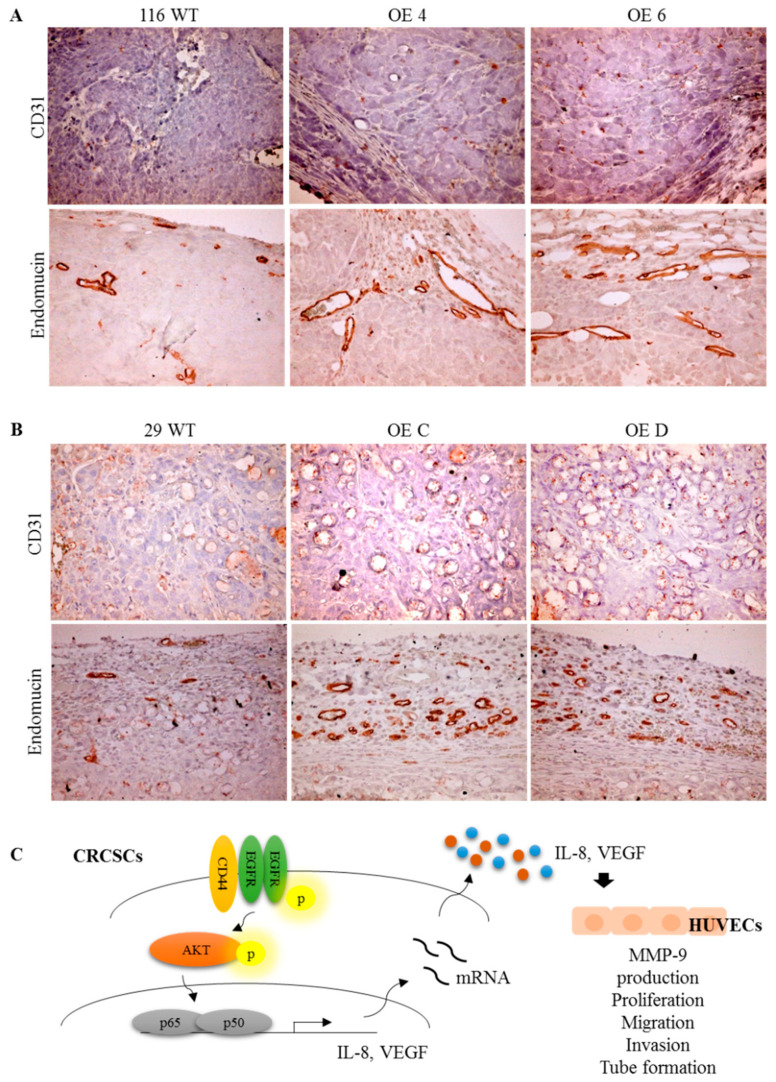
GATA6-overexpressing human CRC cells induce a stronger angiogenesis in vivo. The levels of two endothelial markers, CD31 and endomucin, in the tissues resected from the tumors grown from the parental (WT) and the GATA6-overexpressing clones derived respectively from HCT-116 (**A**) and HT-29 (**B**) cells transplanted into nude mice were analyzed by IHC staining using antibodies against CD31 and endomucin, respectively. (**C**) A proposed model of the angiogenesis-stimulatory effect of the stemness-high GATA6-overexpressing human CRC cells.

To further investigate whether the GATA6-overexpressing human CRC cells could promote angiogenesis in vivo, immunohistochemical (IHC) staining was performed using tissue samples resected from the tumors grown from the clones as well as their parental counterparts transplanted subcutaneously into nude mice by using antibodies against CD31 and endomucin, two EC markers, as probes, respectively. As shown in Figure 6A,B not only were the intensities of both CD31 and endomucin staining signals but also the size and density of the vessels were markedly increased in the tumors grown from the GATA6-overexpressing clones. These observations further supported that the stemness-high human CRC cells induce a stronger in vivo angiogenesis.

## 4. Discussion

Cancer stem cells (CSCs) have been shown to enhance tumor neovascularization by expressing various angiogenic factors which in turn also contributes to their own maintenance and proliferation [58]. For example, significant increases in the migration and tube formation of endothelial cells (ECs) treated with the conditioned media (CM) collected from the stem cell-like glioma cells (SCLGCs) compared with those from the non-SCLGCs were observed which could be accounted by a higher VEGF level in the former [59]. In addition, CSC-enriched spheroid cells grown from both HCT-116 and HT-29 human CRC lines could promote angiogenesis of endothelial progenitor cells (EPCs) through upregulating VEGF expression [60]. Furthermore, CRC-derived MSCs (CRC-MSCs) isolated from primary human CRC tissues could enhance the stemness and the production of VEGF from human CRC cells and the CM prepared from them also increase the in vitro angiogenesis of ECs [61].

Since our previous work has shown that the stemness properties of both HCT-116 and HT-29 human CRC cells were markedly increased by GTAT6 overexpression [43], we postulated that these stemness-high human CRC cells might promote stronger angiogenesis of human ECs. To test this hypothesis, we compared the angiogenesis-promoting effects between the conditioned media (CM) collected from the GATA6-overexpressing HCT-116 and HT-29 clones and their respective vector-control clones. After assuring that the stemness properties of these clones were not significantly affected by a short-term incubation in low serum condition (Figure 1A), we treated HUVECs with CM collected from the clones and found that CM collected from the GATA6-overexpressing human CRC clones induced marked increases of the tube formation, migration, invasion and even DNA synthesis in HUVECs (Figure 1B–E). These results strongly suggested that the stemness-high human CRC cells produce (and secret) more angiogenic factors. Interestingly, elevated mRNA levels (Figure 2A) and secretion (Figure 2B) of MMP-9 were detected in HUVECs after they were incubated with the CM collected from the GATA6-overexpressing human CRC clones which was critical for promoting the tube formation of ECs (Figure 2C). Above findings indicated that MMP-9 produced by HUVECs is essential for their own tube formation as this protease has been reported to not only could enzymatically modify several ECM components which upon cleavage might provide a scaffold for the sprouting endothelium but also release certain sequestered proangiogenic growth factors (e.g., VEGF and bFGF) or directly generate cleaved products with proangiogenic activity [62]. To no surprise, significant increases in the mRNA levels of several pro-agiogenic factors (e.g., VEGF-A, ANG1 and SDF-1) as well as several cytokines (e.g., IL-1β, IL-6, IL-8, TNF-α, and M-CSF) were found in the GATA6-overexpressing clones (Figure 3A,B). In accordance, marked elevations of IL-6, IL-8, and VEGF-A were also detected in the CM collected from these clones, respectively (Figure 3C) and the contributions of the latter two cytokines in the angiogenesis-promoting effects of the CM was verified by the suppression of the CM-induced tube formation of HUVECs by either a small-molecule VEGFR2 inhibitor (Figure 3D) or an IL-8 neutralizing antibody (Figure 3E). While VEGF, particularly VEGF-A, is the main stimulating factor for tumor angiogenesis which acts by binding to the VEGFR2 on ECs and subsequently activating several pathways such as PI3K, PKC, and Ras/Raf/ERK/MAPK [63,64], IL-8 (aka CXCL8) enhances angiogenesis by interacting with the CXCR1/CXCR2 on ECs, resulting in the enhancement of their proliferation, chemotaxis, survival, and protease (MMP) activation [65]. Moreover, IL-8 has recently been shown to increase not only the migration and tube formation of HUVECs but also the mRNA and protein levels VEGF-A, VEGFR1, and VEGFR2 in these cells [66], suggesting that the angiogenesis-promoting effect of IL-8 might be via activating the VEGF-A/VEGFR2 pathway which could be the reason why the tube formation-promoting effects of the CM collected from the GATA6-overexpressing clones on HUVECs were abolished completely by blocking either VEGF-A or IL-8 (Figure 3D,E).

With respect to the signaling pathway(s) responsible for the increased production of VEGF-A and IL-8 in the GATA6-overexpressing human CRC clones, we assessed the involvement of NF-κB pathway because this transcription factor has previously been shown to stimulate the production of a variety of proangiogenic factors including VEGF-A and IL-8 [67]. Indeed, marked increases of the active NF-κB molecules were detected in these clones (Figure 4A,B). More importantly, not only were the mRNA levels of VEGF-A and IL-8 in the clones (Figure 4D) but also the tube formation-stimulatory effects of the CM collected from them (Figure 4E) were dramatically reduced by the treatment of PDTC, a well-known inhibitor of NF-κB (Figure 4C). Subsequently, we found that activation of NF-κB in the GATA6-overexpressing human CRC clones was dependent mainly on the EGFR/AKT pathway (Figure 5) which could be attributed to the dramatic increases of both the total and surface levels of CD44 in these clones [43] because this CRCSC marker was well documented as a co-receptor in the EGFR signaling pathway [68,69,70]. Finally, a stronger in vivo angiogenesis induced by the GATA6-overexpressing human CRC clones was also observed (Figure 6A,B). Together, our findings demonstrate that a stronger angiogenesis-stimulatory effect of the CM collected from the stemness-high GATA6-overexpressing human CRC cells is due mainly to the presence of higher amounts of two proangiogenic factors, VEGF-A and IL-8, whose production is stimulated by the EGFR/AKT/NF-κB pathway (Figure 6C). However, mechanisms such as forming blood vessels directly via transdifferentiating into ECs, vasculogenic mimicry (VM) [71], or by secreting more extracellular vehicles (EVs) such as exosomes [72] have also been reported to be adopted by the CRCSCs for maintenance of cancer vascular niche which might also be worthy of investigation.

## Data Availability

The data that support the findings of this study are available from the corresponding author upon reasonable request.

## Supplementary Materials

The following are available online at https://www.mdpi.com/1422-0067/22/3/1355/s1.
