# Supplementary material for: The Stemness-High Human Colorectal Cancer Cells Promote Angiogenesis by Producing Higher Amounts of Angiogenic Cytokines via Activation of the Egfr/Akt/Nf-κB Pathway
Source: Int J Mol Sci. 2021 Jan 29;22(3):1355. doi: 10.3390/ijms22031355 (PMC7866396; doi:10.3390/ijms22031355)
Supplement: Supplementary file 1 [file ijms-22-01355-s001.pdf]

Supplementary Materials

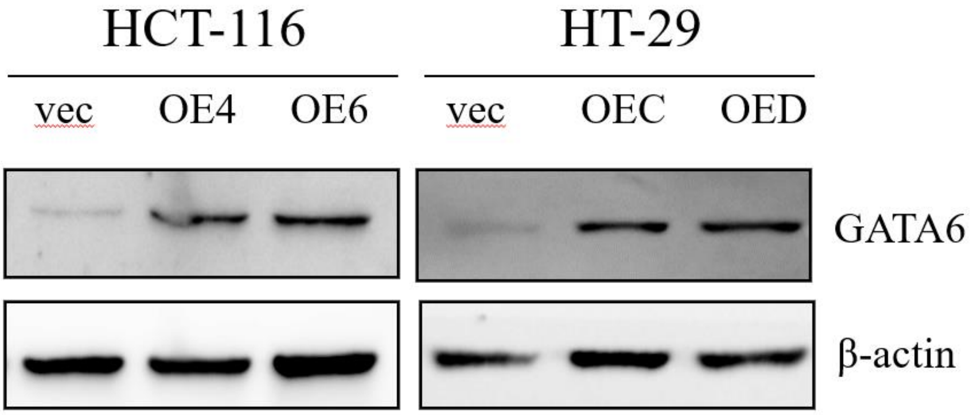

Figure S1. The protein levels of GATA6 in the GATA6-overexpressing HCT-116- and HT-29 clones, respectively. Total lysates (20 μg) prepared from the vector- and GATA6-overexpressing HCT-116 and HT-29 clones, respectively, were subjected to western blot analyses using antibodies against GATA6. β-actin signals were used as loading controls.
